# The importance of studying all subgoals at once

**DOI:** 10.3389/fnhum.2014.00889

**Published:** 2014-11-03

**Authors:** Ludovic Marin

**Affiliations:** Movement to Health Laboratory (M2H), EuroMov, Montpellier-1 UniversityMontpellier, France

**Keywords:** motor control, dynamical systems theory, subgoal, wavelet transform, movement

In Schmidt et al.'s article, an important point has been raised when stating that “*system components are related to different time scales related to subgoals/events*.” Such a claim should lead several authors in the field of motor control to analyze and interpret their outcomes differently. When performing an action, several subgoals are present and have their own role in the general outcome. For instance, when dancing with someone, each dancer has to move his/her own feet at a precise moment to coordinate with the other. At the same moment, hips and trunks could also require specific movements. At a more macroscopic scale, the dancers have to perform a tour (turn), which also imposes a specific action and at an even higher scale, the entire dance situation could have a purpose of seduction, winning a dance competition, boasting and so on. Each of these scales is deeply related and independent at the same time. All scales are related because to achieve a tour for instance, specific foot movements have to be executed. The tour and the foot movements are totally intertwined. But paradoxically they are also independent from each other. If one dancer produces a bigger step than expected, even if at the “tour scale” it will not be observed, at the “feet scales,” the two dancers will have to adapt and modify the further steps to smoothly perform the tour.

If this entire description seems obvious, in reality, researchers do not analyze their data at the level of each subgoals. The usual way of analyzing this dance example would be to subjectively focus on one goal/subgoal and voluntary ignore the others. Consequently some researchers would just focus on the dynamic of the tours, whereas some others would only analyze the feet moves. Based on the purpose of the research, focusing on only one scale is obvious and necessary. However, as stated previously, all scales are intertwined. Is it sufficient to study the dynamic of the tour without analyzing the feet? We think that if researchers try to apprehend the entire complexity of the dance (or any other action), all subgoals have to be taken into account.

Such a whole aspect consideration requires addressing two main issues. First, is there a method able to analyze several time scales of the same action? Second, is there a way to determine what are the functional subgoals?

First, analysis method. The authors used the wavelet (WT) and the cross wavelet transform (CWT) methods (Flandrin, 1988; Torrence and Compo, 1998; Issartel et al., 2006, 2007). WT is suited to analyze one signal, while CWT is based on the common analysis of several signals. The WT/CWT's methods are based on a time-frequency representation that allows the three main components (amplitude, frequency, and phase) of a non-stationary signal to be represented. In our everyday life, movements are non-stationary. For instance if we used the dance situation previously described, the movements of the feet and the dynamic of the tour involve different directions with different frequencies, speed and amplitudes. The WT/CWT were developed to analyze these kinds of signals. Moreover, when one tries to explore several subgoals, no one knows what to analyze in each signal. What to filter, which frequency is functional and which one can be erased and so on. In the WT/CWT methods, there is no need to filter or make a priori subjective choices on frequencies. The outcome of these methods gives figures of the entire frequency spectrum over the time, which immediately reveals the significant parts of the signal. Another advantage of these methods is the “different time scales investigations.” One can obtain an illustration of the entire frequency evolution of the feet (with the WT method) or even cross-frequency and cross-phase (called also relative phase) evolution over the time between the feet and the tour, or even between more than two signals. In other words, such a CWT method is able to reveal what is happening for each subgoal in term of frequency, amplitude and phase over the time.

Second, definition of the subgoals. In the dance example, we described the tour, the feet… but are they real subgoals? And more important, how one can determine a subgoal? Such questions are the most difficult to address. In the literature some researchers are working on determining motor primitive (e.g., Ijspeert et al., 2013) or discriminating in each signal several (sub)segments (e.g., Noy et al., 2011) but these studies are not designed to define subgoals since they are focused on a “micro” level. Subgoals are not just different type of the feet movements, but rather specific purposes at different scales (Varela, 1984). A dance tour has a specific purpose as well as a jump for instance; both are subgoals in the entire choreography. In situated action, an interview-based study, Varela (1984) described how to define goals and subgoals of any action/event. Based on this macroscopic analysis, one might assess the motor evolution of each subpart. However, one can immediately see the problem of mastering the situated action as well as the analyses of motor control. Therefore, before one general method could be created, we suggest using the WT/CWT methods as Schmidt et al. did to address the question of subgoal discrimination. First the WT/CWT bring to light the main significant frequencies and consequently the main range of frequencies to analyze. These frequency ranges will constitute all parts of the signal of particular interest. Then, they used such a technique to reveal different time scale (short and long), all, crucial for understanding the whole aspect of the action observed. Consequently, it seems reasonable to define subgoals by using the WT/CWT by deciphering the functional scales of a particular action/event.

In conclusion, although further studies are necessary to define subgoals, it seems of particular interest to change the way researchers study their action/event by apprehending the entire aspect of each related subgoal of a specific action/event. This will open a new perspective on understanding a particular action.

## Conflict of interest statement

The author declares that the research was conducted in the absence of any commercial or financial relationships that could be construed as a potential conflict of interest.
